# Delayed reorganisation of F-actin cytoskeleton and reversible chromatin condensation in scleral fibroblasts under simulated pathological strain

**DOI:** 10.1016/j.bbrep.2022.101338

**Published:** 2022-09-13

**Authors:** Petar Markov, Hanxing Zhu, Craig Boote, Emma J. Blain

**Affiliations:** a School of Biosciences, Cardiff University, Cardiff, CF10 3AX, UK; b School of Optometry and Vision Sciences, Cardiff University, Cardiff, CF24 4HQ, UK; c School of Engineering, Cardiff University, Cardiff, CF24 3AA, UK; d Biomechanics and Bioengineering Centre Versus Arthritis, School of Biosciences, Cardiff University, Cardiff, CF10 3AX, UK

**Keywords:** Sclera, Fibroblasts, Cytoskeleton, Nucleus, Chromatin condensation parameter, Tensile strain, Glaucoma

## Abstract

Mechanical loading regulates the functional capabilities of the ocular system, particularly in the sclera (‘white of the eye’) – the principal load-bearing tissue of the ocular globe. Resident fibroblasts of the scleral eye wall are continuously subjected to fluctuating mechanical strains arising from eye movements, cerebrospinal fluid pressure and, most influentially, intra-ocular pressure (IOP). Whilst fibroblasts are hypothesised to actively participate in scleral biomechanics, to date limited information has been reported on how the macroscopic stresses and strains are transmitted via their cytoskeletal networks. In this study, the effect of applying either a ‘*physiological load*’ (simulating healthy IOP) or a ‘*pathological load*’ (simulating an elevated glaucomatous IOP) to bovine scleral fibroblasts, as a model of human glaucoma, was conducted to characterise cytoskeletal organisation, chromatin condensation and cell dimensions using immunofluorescence confocal microscopy. Quantification of cell parameters and cytoskeletal element anisotropy were subsequently performed using FibrilTool, and chromatin condensation parameter assessment through a bespoke MATLAB script. The novel findings suggest that physiological load-induced F-actin rearrangement is transient, whereas pathological load, recapitulating *in vivo* glaucomatous IOP levels, had a reversible and inhibitory influence on remodelling of the cytoskeletal architecture and, further, induction of chromatin condensation. Ultimately, this could compromise cell behaviour. These findings could provide valuable insight into the mechanism(s) used by scleral fibroblasts to mechanically adapt to support biomechanical tissue integrity, and how it could be potentially modified for therapeutic avenues targeting mechanically mediated ocular pathologies such as glaucoma.

## Highlights

• Physiological strain induced a transient F-actin rearrangement in scleral fibroblasts.
• In contrast, pathological strain reversibly delayed F-actin rearrangement.
• Vimentin and β-tubulin networks were largely unaffected by strain regimens.
• Pathological strain reversibly increased chromatin condensation parameter.
• Pathological strain may induce ‘inhibition delay’ to confer cytoprotection.

## Graphical abstract

**Figure:**
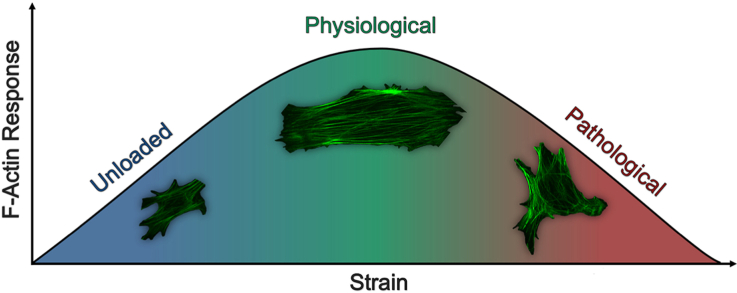

## 1 Introduction

Mechanical loading is important in regulating the functional capabilities of the ocular system via adaptation at both the cell and tissue level. The sclera (‘white of the eye’) is the principal load-bearing tissue of the ocular globe, comprising predominantly of densely woven type I collagen fibril bundles, which give the tissue its opacity and the mechanical rigidity to maintain eye shape and structural integrity [1]. The scleral region immediately adjacent to the optic nerve head (ONH), the peripapillary sclera (PPS), is subjected to significant dynamic forces arising from eye movements, the cerebrospinal fluid pressure and, most influentially, the IOP [2]. Thus, the resident fibroblasts of the scleral eye wall, responsible for synthesis and remodelling of the tissue's extracellular matrix (ECM), are constantly subjected to fluctuating mechanical strains that can trigger tissue level biomechanical changes either via ECM remodelling or via dynamic mechano-signalling processes involving the cytoskeleton [3,4].

The cytoskeleton persists in a state of dynamic equilibrium with constant polymerisation and depolymerisation, a feature which permits regulation of cell stiffness and connectivity to the ECM and adjacent cells. Whilst fibroblasts are hypothesised to actively participate in scleral biomechanics, limited information has been reported to date on how the macroscopic stresses and strains are transmitted via their cytoskeletal networks. The ability to study cytoskeletal remodelling in response to applied loading is fundamental for understanding how deformations may damage cells and tissues and contribute to ocular pathologies with a biomechanical aspect, such as glaucoma [5,6] – the leading cause of irreversible blindness worldwide. Glaucoma initially presents as degradation in the peripheral visual field, progressing centrally, and can lead to blindness if untreated. Glaucoma is classified as a group of optic neuropathies resulting in the loss of retinal ganglion cells (RGCs), along with atrophy and excavation of the optic nerve head [7]. Elevated IOP is a major risk factor for glaucoma [8] and, therefore, an improved understanding of how the scleral fibroblast cytoskeletal components respond to pathological loads could provide valuable insight into the mechanism(s) used by these cells to mechanically adapt to environmental changes.

The objective of the current study was to characterise early responses in the reorganisation of the three principal cytoskeletal elements in a bovine model of human glaucoma whereby scleral fibroblasts were subjected to either a ‘*physiological load*’, simulating healthy IOP or a ‘*pathological load*’, simulating an elevated glaucomatous IOP.

## 2 Materials and methods

All reagents were obtained from Sigma-Aldrich (Poole, UK) unless specified otherwise.

### 2.1 Bovine scleral fibroblast isolation and culture

Scleral fibroblasts were obtained, using an explant crawl-out approach, from 20 bovine eyes (mean age of 23.4 ± 4.4 months) within 12 h post-mortem (Maddock Kembrey Meats Lt abattoir, Maesteg, UK) as previously described [9]. Ethical approval was not required as the tissue was a by-product of the food industry. Cells were seeded at a density of 4 × 10^5^ fibroblasts per well of a BioFlex™ type I collagen coated 6-well culture plate (Catalogue # BF–3001C; Dunn Labortechnik, Asbach, Germany) in Dulbecco's Modified Eagle's Medium (DMEM)/Hams F12 with Glutamax™ (containing 100 μg/ml penicillin, 100U/ml streptomycin, 50 μg/ml ascorbate-2-phosphate and 10% (v/v) foetal calf serum) and stabilised for 24 h prior to loading.

### 2.2 Application of mechanical strain

Using the FX3000 system (Flexcell International®, Burlington, USA), an equibiaxial strain was applied which reflects the circumferential (in-wall) hoop stress experienced *in vivo* by fibroblasts resident in the peri-peripapillary sclera [10]. Physiological (normal IOP) and pathological (elevated IOP, typically observed in glaucoma) strains were calculated based on reported data of average baseline bovine eye IOP of 27mmHg (3.6 kPa) and 60mmHg (8 kPa), respectively [11,12]. The Young-Laplace equation (σ = pr/2t where σ = in-wall stress; p = IOP; r = eye radius; t = eye tunic thickness) which defines the stress as an applied force from the IOP acting normal to the inner surface of the scleral wall per m^2^ of tissue cross-sectional area, was utilised to determine the IOP-induced hoop stress (and hence target strain) after approximating the eye globe shape to a sphere, using an average bovine eye wall thickness of 1.6 mm, a radius of 16.2 mm and Young's modulus range of 1–7 MPa [13] (capturing the strain-stiffening response of the bovine sclera with IOP, typical of collagenous connective tissues as fibrils are recruited under loading) [14]. Hence, bovine scleral fibroblasts were either subjected to a physiological cyclic tensile strain (CTS) of between 0.26% and 1.8% (deriving from an average IOP of 27mmHg over a 1–7 MPa Young's modulus variation), or a pathological CTS of between 0.6% and 4% (deriving from an average IOP of 60mmHg over the same modulus range). For both regimens the strain range was temporally modulated at a frequency of 1Hz, representative of the physiological ocular pulse frequency [15]. Unloaded cells served as controls. CTS was globally applied to all cells for 1 h using the FX3000 system at 37 °C, and scleral fibroblasts fixed at 1 h, 6 h and 24 h after CTS ceased using 2% (w/v) paraformaldehyde (15min).

### 2.3 Immunohistochemical visualisation of cytoskeletal elements

Scleral fibroblasts were labelled for F-actin (Alexa-488™ phalloidin (1:40 dilution); Invitrogen, UK), β-tubulin microtubules (mouse anti-tubulin E7 antibody (1:500 dilution; Developmental Studies Hybridoma Bank, USA) and vimentin intermediate filaments (mouse anti-vimentin V9 antibody; 1:100 dilution) prior to mounting in Vectashield™ antifade medium containing 1.5 μg/ml DAPI (Vector Laboratories, USA) as previously described [9,16]. Membranes were divided into segments by cutting, sections were co-labelled for vimentin and F-actin and separately labelled for β-tubulin and representative cells taken from the same well for consistency. Cytoskeletal structures were imaged using a Zeiss LSM880 Airyscan™ upright laser scanning confocal microscope operated through Zen Black software (Carl Zeiss Ltd, Germany). Image acquisition was performed using a Plan-Apochromat 63x/1.4 Oil DIC M27 oil immersion objective lens with laser line switching used for sequential scanning of DAPI (Ex max: 358 nm; Em max: 461 nm) and Alexa 488 (Ex max: 495 nm; Em max: 520 nm) in Airyscan Fast mode. Z-stacks were taken using 2x Nyquist sampling resulting in a Z-step of 0.14 μm (average of 32 optical slices per Z-stack) and reconstructed into 3D isosurfaces using Imaris 9.2 (Bitplane, Zurich, Switzerland). Representative cells were imaged across three different wells per plate (n = 3 wells) and across three independent experiments (N = 3) accounting for a total of 103–154 cells per label per regime i.e. for each time point and strain amplitude applied.

### 2.4 Quantification of cell and cytoskeletal element parameters

The FIJI distribution of ImageJ [17] was used to quantitatively assess cell parameters from Z-axis maximum intensity projections of confocal image stacks; cell and nucleus area were calculated by creating a region of interest around the cortical F-actin and nucleus boundaries, respectively, and assessing total area; cell eccentricity was measured as the ratio between cell width and length, with values closer to 0 signifying a more elongated shape. Cytoskeletal fibre anisotropy was measured with the ImageJ plugin FibrilTool [17,18], with randomly aligned fibres yielding values close to 0 and highly aligned fibres closer to 1. Chromatin condensation parameter (CCP) was calculated through a bespoke MATLAB script as previously described [19]. Based on the Sobel edge detection [20], an objective parametric assessment of chromatin compaction is produced, representative of the ratio between the registered edges in pixels against the total pixel area, permitting comparison between differently sized nuclei and a CCP-based evaluation of chromatin condensation [[21], [22], [23], [24]].

### 2.5 Statistical analysis

Quantitative data is presented as box-whisker plots containing the median and the 25th and 75th percentiles, and the minimum and maximum excluding outlier values. All data were tested for normality (Anderson-Darling test) and equal distribution (Bartlett's two-sample F-test) prior to a one-way analysis of variance (ANOVA; Tukey's *post-hoc* test) in MATLAB 9.6 (MathWorks, Natick, USA). Where data was not normal/equal variance, a non-parametric Kruskal-Wallis test was implemented. Differences were considered significant at p < 0.05 and represented using a bespoke MATLAB script [25].

## 3 Results

### 3.1 Increased cell area in response to cyclic tensile strain

**Figure 1:**
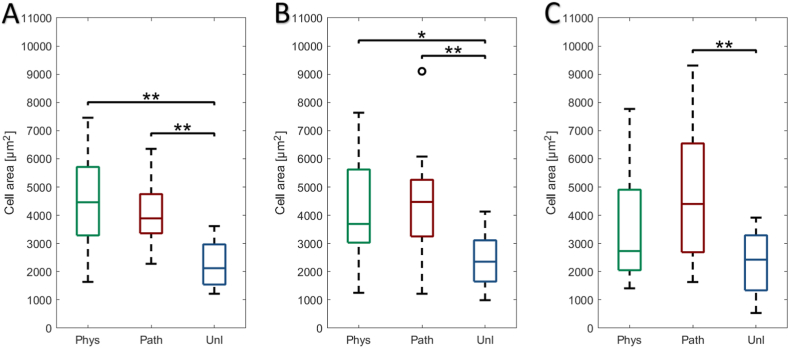
Effect of physiological (0.26–1.8%, 1Hz, 1 h – *‘phys’*) or pathological (0.6–4%, 1Hz, 1 h – *‘path’*) cyclic tensile strain (CTS) on bovine scleral fibroblast cell area at **(A)**1 h, **(B)** 6 h and **(C)** 24 h post-cessation; unloaded cells served as controls (*‘unl’*). Cell area was calculated by region of interest (ROI)-based ImageJ analysis and representative data are presented as median ± standard deviation of 103–145 cells (n = 3 wells, N = 3 plates) [*p < 0.05; **p < 0.01].

Scleral fibroblast cell area increased significantly in response to CTS in comparison to the unloaded cells which did not alter appreciably over the 24 h (Fig. 1). Fibroblast area following 1 h post-cessation of either physiological (p < 0.01) or pathological CTS (p < 0.01), increased by approximately 200% relative to unloaded cells (Fig. 1A). This response was also observed at 6 h post-physiological (p < 0.05) and post-pathological CTS (p < 0.01) relative to unloaded cells (Fig. 1B). After 24 h, cell area was only significantly larger in cells exposed to pathological CTS versus unloaded cells (p < 0.01, Fig. 1C).

### 3.2 Increased cell elongation in response to cyclic tensile strain

**Figure 2:**
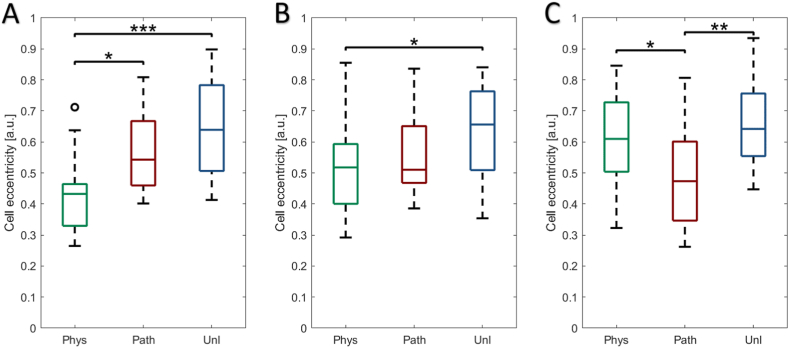
Effect of physiological (0.26–1.8%, 1Hz, 1 h – *‘phys’*) or pathological (0.6–4%, 1Hz, 1 h – *‘path’*) cyclic tensile strain (CTS) on bovine scleral fibroblast cell eccentricity area at **(A)**1 h, **(B)** 6 h and **(C)** 24 h post-cessation; unloaded cells served as controls (*‘unl’*). Cell eccentricity was calculated by region of interest (ROI)-based ImageJ analysis and representative data are presented as median ± standard deviation of 103–145 cells (n = 3 wells, N = 3 plates) [*p < 0.05; **p < 0.01; ***p < 0.001].

A significant increase in cell elongation (i.e. reduction in eccentricity value) was observed 1 h post-physiological CTS compared to both unloaded (p < 0.001) and cells subjected to pathological CTS (p < 0.05, Fig. 2A). Elongated cell shape was still observed 6 h post-cessation of physiological CTS (p < 0.05, Fig. 2B), however by 24 h, cell shape (eccentricity) had returned to that of unloaded cells (Fig. 2C). In contrast, following 24 h recovery from pathological CTS, cell shape was still elongated compared to physiological CTS (p < 0.05) and unloaded cells (p < 0.01).

### 3.3 Increased nucleus area in response to cyclic tensile strain

**Fig. 3:**
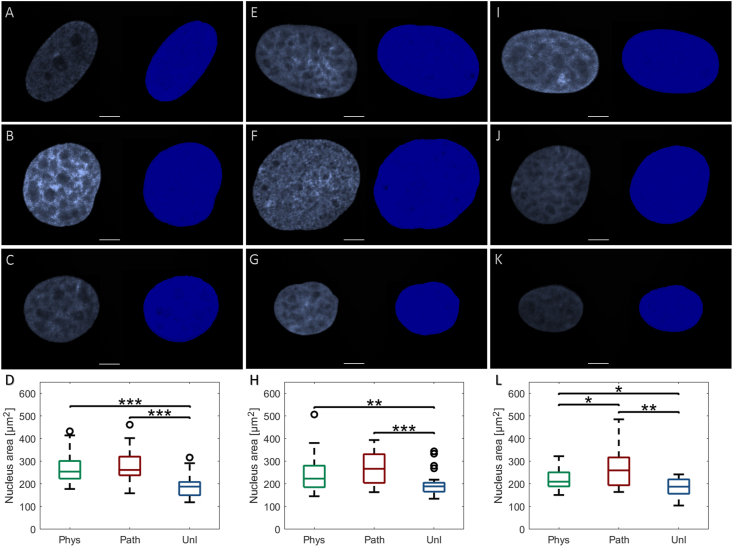
Effect of physiological (0.26–1.8%, 1Hz, 1 h – *‘phys’*) or pathological (0.6–4%, 1Hz, 1 h – *‘path’*) cyclic tensile strain (CTS) on bovine scleral fibroblast nuclei; unloaded cells served as controls (*‘unl’*). Cells were labelled with DAPI, visualised using a confocal microscope and Z-stack maximum intensity projections reconstructed to measure nuclear area using ROI-based ImageJ analysis. Representative nuclei with corresponding Imaris surface reconstructions visualised at 1 h following application of **(A)***phys*, **(B)***path* or **(C)***unl*, **(D)** quantitative measurements; at 6 h following application of **(E)***phys*, **(F)***path* or **(G)***unl*, **(H)** quantitative measurements; at 24 h following application of **(I)***phys*, **(J)***path* or **(K)***unl*, **(L)** quantitative measurements. Quantitative data are presented as median ± SD and are representative of 227–299 nuclei (n = 3 wells, N = 3 plates). [*p < 0.05; **p < 0.01; ***p < 0.001] (scale bar = 10 μm).

Scleral fibroblast nucleus area increased significantly in response to CTS in comparison to the unloaded cells, which did not alter appreciably over the 24 h (Fig. 3). Both physiological and pathological CTS significantly increased nucleus area following 1 h (p < 0.001 and p < 0.001 respectively; Fig. 3A and B), 6 h (p < 0.01 and p < 0.001 respectively; Fig. 3E and F) and 24 h post-CTS cessation (p < 0.05 and p < 0.01 respectively; Fig. 3I and J).

### 3.4 Pathological CTS reversibly increases chromatin condensation parameter

**Fig. 4:**
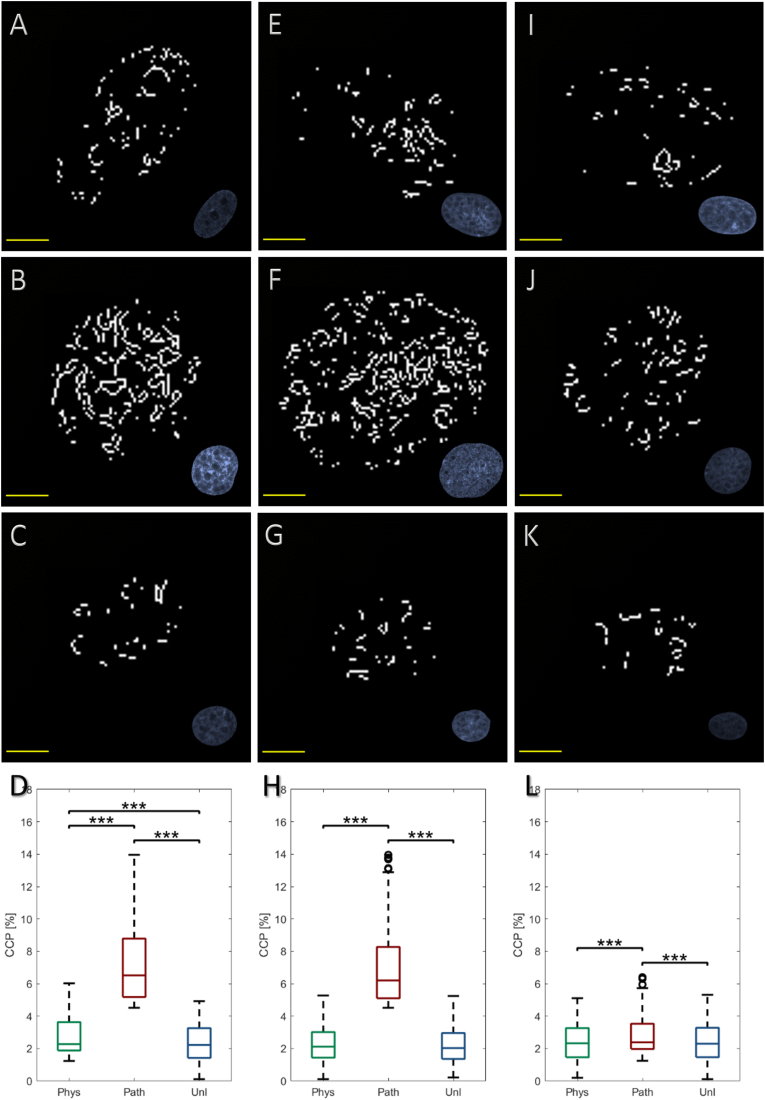
Effect of physiological (0.26–1.8%, 1Hz, 1 h – *‘phys’*) or pathological (0.6–4%, 1Hz, 1 h – *‘path’*) cyclic tensile strain (CTS) on bovine scleral fibroblast nuclear chromatin condensation; unloaded cells served as controls (*‘unl’*). Cells were labelled with DAPI, visualised using a confocal microscope and Z-stack maximum intensity projections reconstructed to measure chromatin condensation parameter (CCP) using a bespoke MATLAB script [16]. Representative algorithm edge detected nuclei visualised at 1 h following application of **(A)***phys*, **(B)***path* or **(C)***unl*, **(D)** quantitative measurements; at 6 h following application of **(E)***phys*, **(F)***path* or **(G)***unl*, **(H)** quantitative measurements; at 24 h following application of **(I)***phys*, **(J)***path* or **(K)***unl*, **(L)** quantitative measurements. Quantitative data are presented as median ± SD and are representative of 227–299 nuclei (n = 3 wells, N = 3 plates). [*p < 0.05; **p < 0.01; ***p < 0.001] (scale bar = 10 μm).

Chromatin condensation, as represented by CCP, was observed in all fibroblast nuclei (CCP>0), however, this was lowest in unloaded cells, and did not alter with time (Fig. 4C, G, K). Significant differences following physiological CTS were only observed at 1 h, with a 21% increase in CCP compared to unloaded cells (p < 0.001; Fig. 4A, D), reverting to basal levels by 6 h (Fig. 4E, H). In contrast, CCP was significantly elevated in cells subjected to pathological CTS and analysed at 1 h (310%, p < 0.001; Fig. 4B, D), 6 h (298%, p < 0.001; Fig. 4F, H) and 24 h post-cessation (22%, p < 0.001; Fig. 4J, L) compared to unloaded cells; this differential response was also significant relative to cells exposed to physiological CTS. However, following 24 h, CCP had begun to return to basal levels, in line with the other experimental groups.

### 3.5 Physiological CTS induces early reorganisation of F-actin stress fibres, whereas response is delayed in response to pathological CTS

**Fig. 5:**
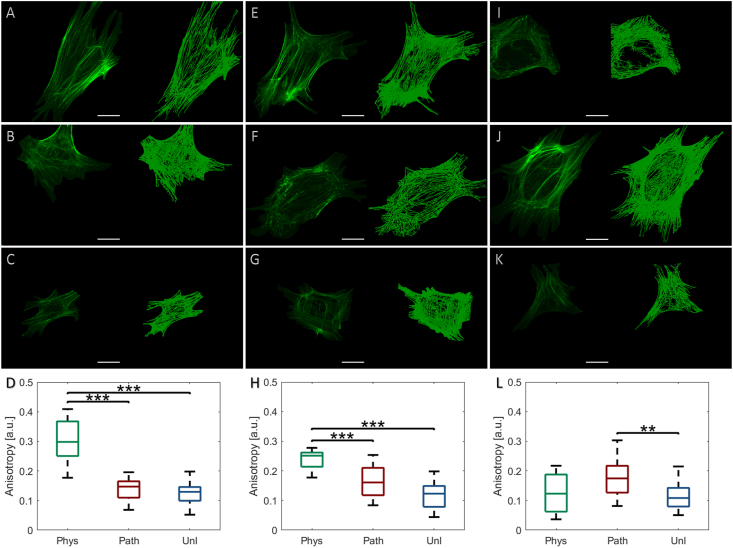
Effect of physiological (0.26–1.8%, 1Hz, 1 h – *‘phys’*) or pathological (0.6–4%, 1Hz, 1 h – *‘path’*) cyclic tensile strain (CTS) on bovine scleral fibroblast F-actin organisation; unloaded cells served as controls (*‘unl’*). Cells were labelled with Alexa-488™ phalloidin, visualised using a confocal microscope and Z-stack maximum intensity projections reconstructed to measure F-actin stress fibre anisotropy using FibrilTool. Representative cells with corresponding Imaris surface reconstructions were visualised at 1 h following application of **(A)***phys*, **(B)***path* or **(C)***unl*, **(D)** quantitative measurements; at 6 h following application of **(E)***phys*, **(F)***path* or **(G)***unl*, **(H)** quantitative measurements; at 24 h following application of **(I)***phys*, **(J)***path* or **(K)***unl*, **(L)** quantitative measurements. Quantitative data are presented as median ± SD and are representative of 103–145 cells (n = 3 wells, N = 3 plates). [**p < 0.01; ***p < 0.001] (scale bar = 25 μm).

One hour following application of physiological CTS, the F-actin cytoskeleton extensively remodelled, with greater elongation and alignment of actin stress fibres (Fig. 5A), when compared to the unloaded group (Fig. 5C). In contrast, F-actin distribution remained largely isotropic in fibroblasts analysed 1 h after cessation of pathological CTS (Fig. 5B), with a mostly rounded cell morphology, reminiscent of the unloaded cells. When F-actin distribution was quantified, significantly greater F-actin stress fibre anisotropy was observed in fibroblasts 1 h after exposure to physiological strain compared to both the unloaded and pathologically loaded cells (p < 0.001; Fig. 5D). Following 6 h post-CTS cessation, F-actin retained a heightened alignment in cells exposed to physiological CTS, although this effect appeared to be less pronounced than that observed at 1 h post-CTS (Fig. 5E); when quantified, F-actin anisotropy had significantly decreased in cells at 6 h post-physiological CTS compared to 1 h (p < 0.05) but retained a noticeable significance relative to the two other groups (Fig. 5H). In contrast, pathologically strained cells displayed a larger F-actin anisotropy than that observed at 1 h (0.142 ± 0.037 at 1 h and 0.165 ± 0.053 at 6 h post-CTS), however, the greater alignment was not significantly different from that of the corresponding unloaded fibroblasts (Fig. 5H). Unloaded scleral fibroblasts did not display any noticeable changes from those exhibited at 1 h (Fig. 5G). At 24 h after cessation of CTS, the morphology of the fibroblasts exposed to physiological strain (Fig. 5I) was comparable to the unloaded cells (Fig. 5K), with low fibre anisotropy and high eccentricity. However, the remodelled F-actin architecture of the pathologically loaded fibroblasts exhibited significantly increased anisotropy (Fig. 5J and L; p < 0.01) compared to its organisation at 6 h (Fig. 5F); the F-actin organisation visualised at 24 h post-pathological strain (Fig. 5J) was reminiscent of its distribution in cells 1 h after exposure to physiological strain, with a dense network of fibre bundles (Fig. 5A).

**Fig. 6:**
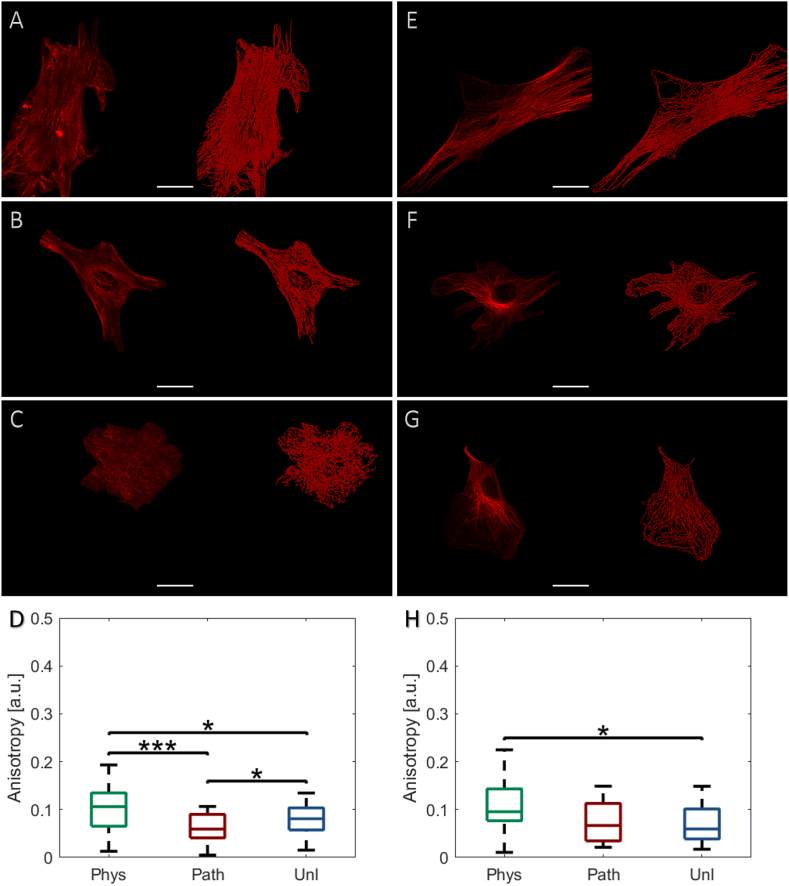
Effect of physiological (0.26–1.8%, 1Hz, 1 h – *‘phys’*) or pathological (0.6–4%, 1Hz, 1 h – *‘path’*) cyclic tensile strain (CTS) on bovine scleral fibroblast **(A**–**D)** vimentin and **(E**–**H)** β-tubulin organisation; unloaded cells served as controls (*‘unl’*). Cells were labelled with V9 (vimentin) or E7 (β-tubulin) primary and Alexa-594™ secondary antibodies, visualised using a confocal microscope and Z-stack maximum intensity projections reconstructed to measure cytoskeletal protein anisotropy using FibrilTool. At 1 h following load application, representative cells with corresponding Imaris surface reconstructions were visualised for vimentin architecture **(A)***phys*, **(B)***path* or **(C)***unl*, **(D)** quantitative measurements, and for β-tubulin architecture **(E)***phys*, **(F)***path* or **(G)***unl*, **(H)** quantitative measurements. Quantitative data are presented as median ± SD and are representative of 103–145 cells (n = 3 wells, N = 3 plates). [*p < 0.05; **p < 0.01; ***p < 0.001] (scale bar = 25 μm).

Interestingly, increased vimentin (Fig. 6A and D; p < 0.05) and β-tubulin (Fig. 6E and H; p < 0.05) anisotropy was only observed at 1 h following physiological CTS, with organisation returning to that observed in unloaded cells prior to the 6 h time point (Supplementary Figs. 1 and 2). A small, but significant reduction in vimentin anisotropy was observed at 1 h following pathological CTS, however this response was transient (Fig. 6B and D; p < 0.05), and was not observed for β-tubulin. Pathological CTS did not significantly influence either vimentin (Supplementary Fig. 1) or β-tubulin anisotropy beyond 1 h (Supplementary Fig. 2).

## 4 Discussion

Under *in vivo* conditions, scleral fibroblasts are exposed to pulsatile IOP, simulated in this study by the applied physiological CTS (0.26–1.8% strain, 1Hz). High tension glaucoma was represented by the elevated pathological CTS (0.6–4% strain, 1Hz), with both experimental regimens never reaching complete relaxation i.e. 0% strain, replicating that observed *in situ*. Physiological loading is known to influence cell shape and cytoskeletal fibre architecture, reflecting the principle of cell ‘tensegrity’ and biomechanical prestress [26,27]. However, although all three cytoskeletal components were observed to contribute to the cellular rearrangement of scleral fibroblasts, the loading regimes utilised primarily induced F-actin stress fibre remodelling with increased vimentin and β-tubulin anisotropy only observed at 1 h following physiological CTS; this corroborates previous observations in bovine chondrocytes [28] and an NIH3T3 fibroblast cell line [29] where there was minimal effect on either the vimentin or microtubule networks in response to mechanical perturbation.

Overall, after removal of physiological CTS, fibroblasts progressively lost their high fibre anisotropy, becoming more isotropic with a gradual realignment of the cell's architecture to their native ‘basal’ state over the 24 h time point. This corroborates previous numerical findings (using finite element modelling) where compressed cells returned to a steady-state, after removal of external forces, due to a time-dependent rearrangement of the F-actin cytoskeleton [30]. As the intermediate filaments have been observed to directly interact with F-actin [31], the fast reorganisation of the vimentin cytoskeleton into a more interwoven network observed after 1 h could be attributed to F-actin remodelling.

Interestingly, following pathological CTS to mimic elevated IOP, the cells initially exhibited disorganised stress fibres, comparable to their previously unloaded state. However, based on the data there is a time-dependent delay in F-actin stress fibre orientation in response to pathological strain, which becomes more structurally organised at 24 post-CTS cessation. Thus, it is hypothesised that the cell phenotype/behaviour following pathological load could be a compensatory protective mechanism for preservation of cell integrity, along with ‘inhibition delay’ of *in situ* cytoskeletal reorganisation. However, inclusion of further time points for analysis, i.e. beyond 24 h, would be required to determine whether cytoskeletal organisation in the pathologically loaded cells return to their basal state at the same rate as the fibroblasts exposed to physiological strain and would elucidate whether it is purely a delayed response and/or a cytoprotective mechanism.

It is widely acknowledged that mechanical forces influence the dynamic assembly of the ‘adhesome’ [32], a complex network that assembles as a consequence of ECM ligand - integrin engagement, resulting in actin cytoskeleton rearrangement and downstream involvement of adaptor proteins, receptors, kinases and phosphatases which control cellular behaviours and tissue homeostasis. Several putative mechanisms have been hypothesised to be involved in fibroblast mechano-sensing, and particularly pertinent to ocular biomechanics is integrin signalling. Activation of α_v_β_3_ integrin has been shown to increase IOP in mice, with deletion of the β_3_ integrin subunit resulting in the significant lowering of IOP [33]. It would be interesting to ascertain whether the delayed reorganisation of the actin cytoskeleton in response to pathological CTS involves alterations in integrin expression and/or activation. Another possible pathway that might be implicated in the observed ‘inhibition delay’ is modulation of RhoA/ROCK signalling that has been reported to induce tenascin-C transcription in chick embryo fibroblasts [34]; this has direct implication as tenascin-C remodelling has been previously reported to be strongly associated with elevated IOP in glaucoma [35]. However, preliminary quantitative PCR analyses indicate that tenascin-C mRNA levels are not significantly regulated under the loading regimens and timeframes utilised in this study, however this putative mechanism warrants further investigation at the protein level.

Although beyond the scope of this study, which utilised loading regimens to simulate acute or early scleral fibroblast mechano-responses, increased loading periods could also be employed to replicate chronically high IOP to ascertain any differential cytoskeletal responses. In most types of glaucoma, elevated IOP generates hydrostatic pressure in conjunction with increased tensile force in the scleral wall, affecting both the ECM and the resident fibroblasts [2]. *In vivo* cellular responses to mechanical load can influence many downstream pathways, following cytoskeletal network reorganisation, as indicated above, potentially affecting functional properties e.g. stiffening of the sclera which impacts on tissue health [36,37].

Moreover, the observation of higher chromatin condensation parameter values [[19], [20], [21],^23^], indicative of greater chromatin condensation which corresponds to more heterochromatin, in scleral fibroblasts exposed to pathological CTS may be suggestive of a gene silencing response due to entry into a quiescent state, and may support the idea of a delayed reaction to confer cyto-protection. Preliminary quantitative PCR analyses has indicated that although transcript levels of the three cytoskeletal proteins are unaffected by the loading regimens, there was a small but significant delay in col1a1 transcription that was rectified by 24 h (data not shown). Further work is required to investigate the relevance of increased chromatin condensation in the scleral fibroblast mechano-response. It has previously been reported that induction of reversible chromatin condensation, arising from epigenetic alterations, in NIH3T3 mouse fibroblasts exposed to static compressive forces was driven by actin depolymerisation [38]; it was hypothesised that this differential transcriptional response observed in these fibroblasts following chromatin condensation permitted maintenance of mechanically regulated tissue homeostasis.

Collectively, these observations would suggest that normal levels of IOP have an integral role in regulating tensional homeostasis due to a rapid but transient remodelling of F-actin stress fibre and chromatin organisation in scleral fibroblasts. Conversely, under elevated IOP conditions a sustained effect was observed which may prevent scleral fibroblasts from assuming the *in situ* cytoskeletal organisation, ultimately preventing normal cellular function which could compromise cell/tissue remodelling if prolonged. This knowledge will further our understanding of scleral cell biomechanical behaviour with a long-term translational goal, beyond the scope of this current study, of using the information to develop novel therapeutic avenues for disorders such as glaucoma.

A caveat of this study is that ‘snapshots’ of cytoskeletal organisation were captured at defined time points post-cessation of CTS in individual cells; however, use of live-cell imaging would be advantageous to visualise the effects of the different loading regimens on the scleral fibroblast cytoskeleton, facilitating the characterisation of remodelling behaviours of each of the cytoskeletal elements in the same cell over time [39]. Furthermore, this study specifically sought to investigate early cellular responses to load application which arguably does not recapitulate the sustained alteration in ocular biomechanics observed with elevated IOP *in vivo*; therefore, increasing the duration of loading will form the basis of future follow-on studies. Finally, the use of Laplace Law in estimating scleral wall hoop stresses is widely acknowledged as being sub-optimal in not fully capturing biologically accurate morphological features of the bovine (and human) eyeball tunic, notably regional variations in wall thickness along with its approximation of eyeball sphericity [40].

## 5 Conclusion

This study was the first to compare organisation of the three principle cytoskeletal components in scleral fibroblasts exposed to either a physiological or pathological strain, representative of *in vivo* IOP experienced in scleral tissue. Whilst it has been extensively documented in human glaucoma and experimental animal models that elevated IOP disrupts the typical scleral ECM organisation [[41], [42], [43], [44], [45]], less is known regarding the scleral fibroblast response. Therefore, these novel findings suggest that pathological CTS, recapitulating *in vitro* the glaucomatous levels of IOP had a reversible and inhibitory influence on remodelling of the F-actin cytoskeletal architecture and induction of chromatin condensation, which may compromise cell behaviour and contribute to glaucomatous pathology.

## Funding

This work was supported by a Doctoral Training Grant from the 10.13039/501100000266EPSRC. The funders were not involved in the study.

## Author contributions

Study conception/design – PM, CB, EJB.

Data acquisition and statistical analysis – PM.

Data analysis and interpretation - PM, CB, EJB.

Manuscript preparation – PM, CB, EJB.

Critical revision of the draft – PM, HZ, CB, EJB.

All authors approved the final manuscript and take full responsibility for integrity of the study.

## Data access statement

Information on the data underpinning the results presented here can be found in the Cardiff University data catalogue at 10.17035/d.2020.0118671701.

## Declaration of competing interest

The authors declare no competing interests.

## Data Availability

We have included link to Cardiff University data catalogue for access to material

## Appendix A Supplementary data

figs1 Effect of physiological (0.26–1.8%, 1Hz, 1h – *‘phys’*) or pathological (0.6–4%, 1Hz, 1h – *‘path’*) cyclic tensile strain (CTS) on bovine scleral fibroblast vimentin organisation; unloaded cells served as controls (*‘unl’*). Cells were labelled with V9 primary and Alexa-594™ secondary antibodies, visualised using a confocal microscope and Z-stack maximum intensity projections reconstructed to measure vimentin intermediate filament anisotropy using FibrilTool. Representative cells with corresponding Imaris surface reconstructions were visualised at 6h following application of **(A)***phys*, **(B)***path* or **(C)***unl*, **(D)** quantitative measurements and at 24h following application of **(E)***phys*, **(F)***path* or **(G)***unl*, **(H)** quantitative measurements. Quantitative data are presented as median ± SD and are representative of 103–145 cells (n = 3 wells, N = 3 plates); (scale bar = 25 μm) figs1

figs2 Effect of physiological (0.26–1.8%, 1Hz, 1h – *‘phys’*) or pathological (0.6–4%, 1Hz, 1h – *‘path’*) cyclic tensile strain (CTS) on bovine scleral fibroblast β-tubulin organisation; unloaded cells served as controls (*‘unl’*). Cells were labelled with E7 primary and Alexa-594™ secondary antibodies, visualised using a confocal microscope and Z-stack maximum intensity projections reconstructed to measure β-tubulin microtubule anisotropy using FibrilTool. Representative cells with corresponding Imaris surface reconstructions were visualised at 6h following application of **(A)***phys*, **(B)***path* or **(C)***unl*, **(D)** quantitative measurements and at 24h following application of **(E)***phys*, **(F)***path* or **(G)***unl*, **(H)** quantitative measurements. Quantitative data are presented as median ± SD and are representative of 103–145 cells (n = 3 wells, N = 3 plates); (scale bar = 25 μm). figs2
