# Supplementary figures and images for: Delayed reorganisation of F-actin cytoskeleton and reversible chromatin condensation in scleral fibroblasts under simulated pathological strain
Source: Biochem Biophys Rep. 2022 Sep 13;32:101338. doi: 10.1016/j.bbrep.2022.101338 (PMC9482111; doi:10.1016/j.bbrep.2022.101338)

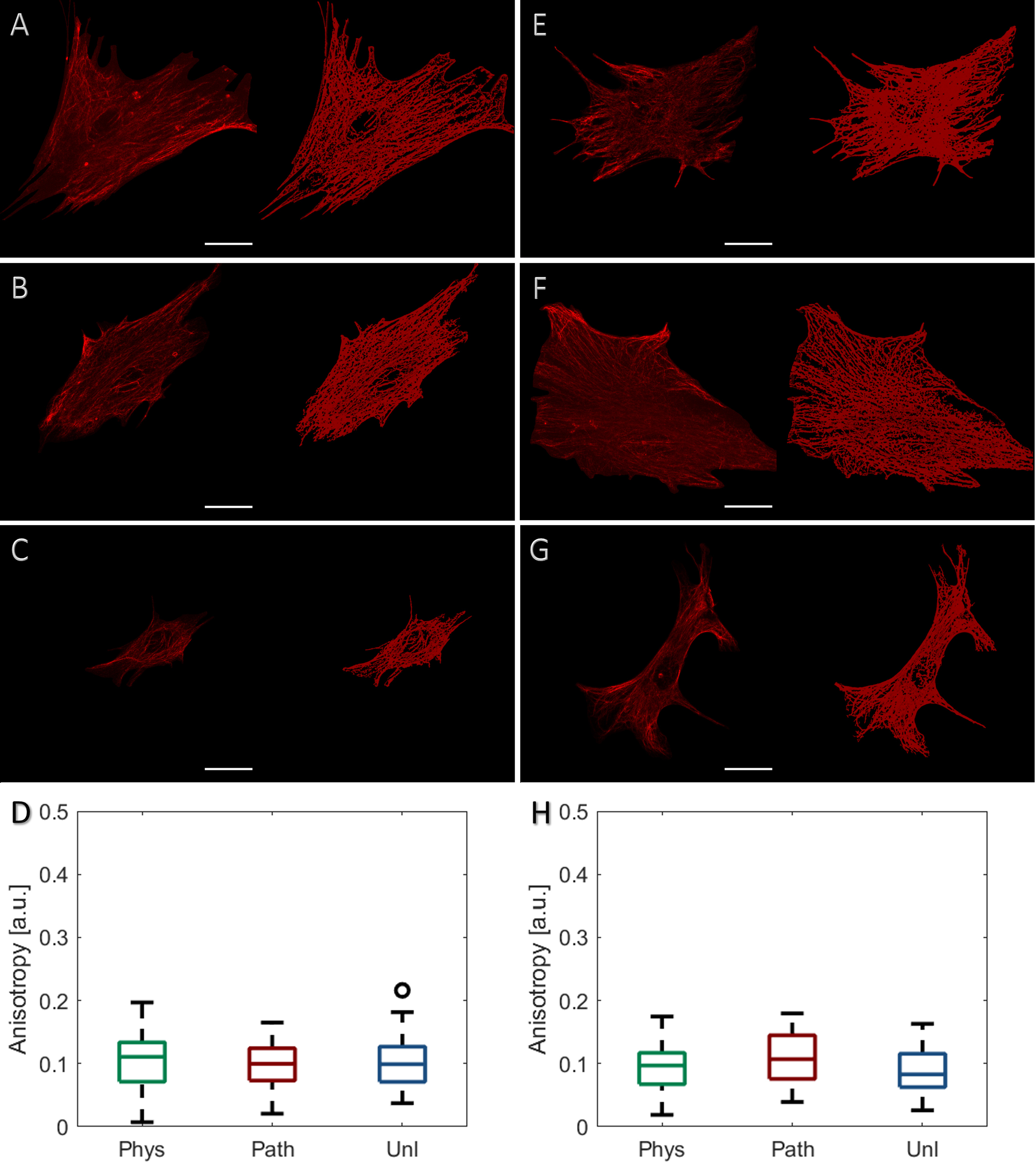

Supplement: figs1 — Effect of physiological (0.26–1.8%, 1Hz, 1h – ‘phys’) or pathological (0.6–4%, 1Hz, 1h – ‘path’) cyclic tensile strain (CTS) on bovine scleral fibroblast vimentin organisation; unloaded cells served as controls (‘unl’). Cells were labelled with V9 primary and Alexa-594™ secondary antibodies, visualised using a confocal microscope and Z-stack maximum intensity projections reconstructed to measure vimentin intermediate filament anisotropy using FibrilTool. Representative cells with corresponding Imaris surface reconstructions were visualised at 6h following application of (A)phys, (B)path or (C)unl, (D) quantitative measurements and at 24h following application of (E)phys, (F)path or (G)unl, (H) quantitative measurements. Quantitative data are presented as median ± SD and are representative of 103–145 cells (n = 3 wells, N = 3 plates); (scale bar = 25 μm) [file mmcfigs1.jpg]

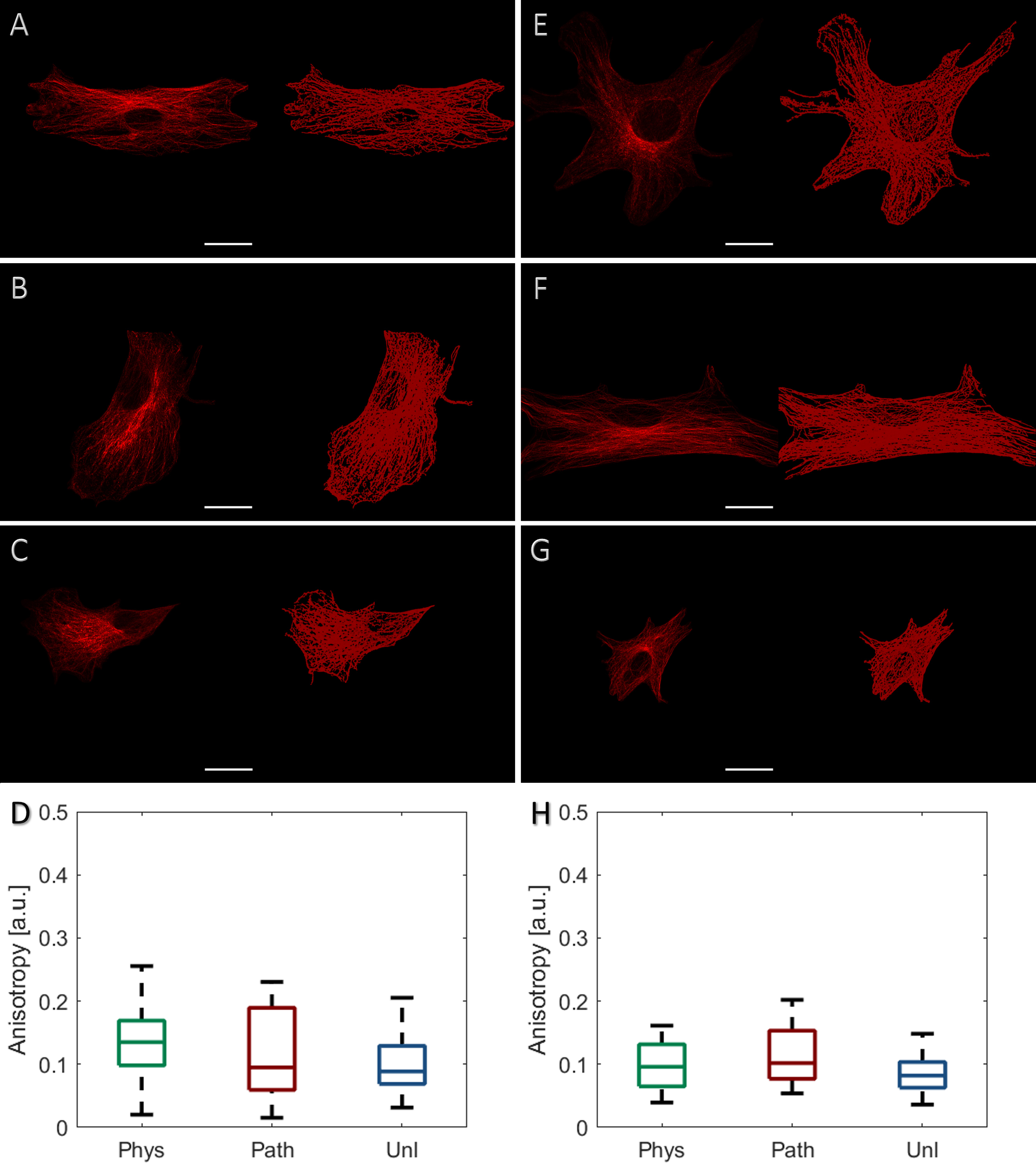

Supplement: figs2 — Effect of physiological (0.26–1.8%, 1Hz, 1h – ‘phys’) or pathological (0.6–4%, 1Hz, 1h – ‘path’) cyclic tensile strain (CTS) on bovine scleral fibroblast β-tubulin organisation; unloaded cells served as controls (‘unl’). Cells were labelled with E7 primary and Alexa-594™ secondary antibodies, visualised using a confocal microscope and Z-stack maximum intensity projections reconstructed to measure β-tubulin microtubule anisotropy using FibrilTool. Representative cells with corresponding Imaris surface reconstructions were visualised at 6h following application of (A)phys, (B)path or (C)unl, (D) quantitative measurements and at 24h following application of (E)phys, (F)path or (G)unl, (H) quantitative measurements. Quantitative data are presented as median ± SD and are representative of 103–145 cells (n = 3 wells, N = 3 plates); (scale bar = 25 μm). [file mmcfigs2.jpg]
